# Virtual ergonomics of PPE systems – a standardization perspective

**DOI:** 10.17179/excli2023-6727

**Published:** 2023-10-27

**Authors:** Peter Bröde, Edith Classen, Jean Léonard, Ronald Heus, Kalev Kuklane

**Affiliations:** 1 Leibniz Research Centre for Working Environment and Human Factors at TU Dortmund (IfADo), Dortmund, Germany; 2 Hohenstein Institute for Textile Innovation GmbH, Bönnigheim, Germany; 3 CENTEXBEL, Grâce-Hollogne, Belgium; 4 Netherlands Institute for Public Safety (NIPV), Arnhem, The Netherlands; 5 Netherlands Institute for Public Safety (NIPV), Zoetermeer, The Netherlands

**Keywords:** Personal protective equipment (PPE), clothing, ergonomics, digital human model, simulation

## ⁯⁯⁯⁯

### Ergonomics of PPE systems

Procedures for testing ergonomics issues concerning the use of personal protective equipment (PPE) have been developed for special groups, e.g. for firefighters involving human participants (Havenith and Heus, 2004[6]). Recently, these procedures gained attention concerning PPE systems, defined as any set of clothing or devices designed to be worn or held by an individual for protection against one or more health and safety hazards. This is corroborated by a European mandate for standardization in this field (European Commission, 2012[4]). A recently published European standard provided procedures for testing the ergonomics of PPE ensembles concerning the mobility, vision, hearing, manual dexterity, and thermal strain of PPE users utilizing human participants (DIN EN 17558, 2023[3]). Nevertheless, there is a need for “*testing methods which are simple, objective and cost-effective. Virtual testing and simulation offer a potential yet under exploited in this area*” (European Commission, 2012[4]). 

### Virtual Ergonomics of PPE systems

**Figure 1:**
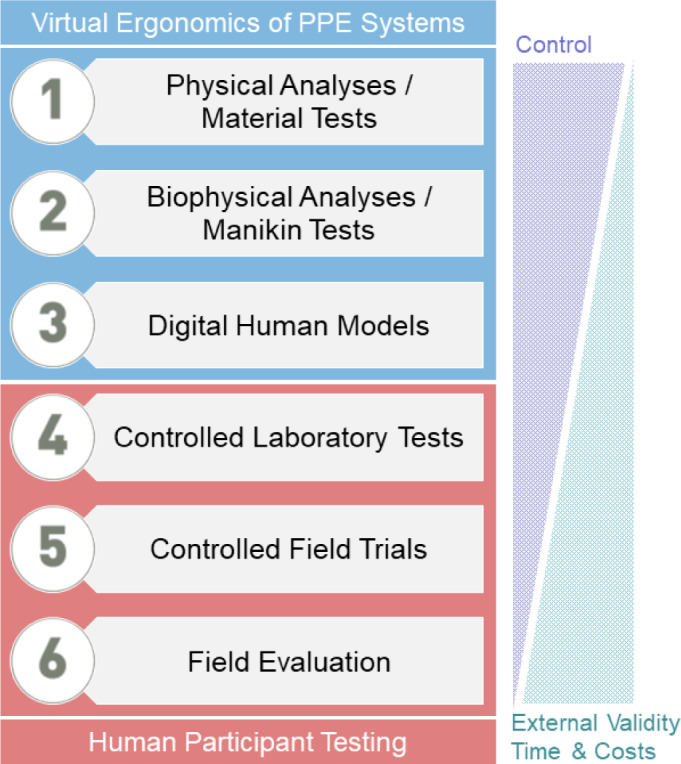
Evaluation levels for the ergonomics of PPE systems, with experimental control decreasing, but external validity as well as costs and time requirements increasing from virtual testing (levels 1-3) to human participant testing (4-6)

Adopting concepts predominantly developed for the thermal aspects in protective clothing design (Goldman, 1974[5]; Potter et al., 2018[13]; Umbach, 1988[16]), Figure 1(Fig. 1) introduces six levels for evaluating the ergonomics of PPE systems. The control of the testing procedure will decrease from level 1 to 6, whilst external validity, but also time requirements and costs will increase. Whereas levels 4 to 6 involve human test persons in the laboratory, in controlled field trials or in observational evaluation studies, level 3 modelling supplemented by physical analyses and material tests (level 1) and biophysical methods using human shaped manikins (level 2) are shaping the area of virtual ergonomics of PPE systems.

Simulation tools employing material and biophysical testing linked with digital modelling may help to develop design solutions to ergonomics issues at reduced costs compared to human participant testing, with numerous applications concerning the thermal strain by PPE use (Awais et al., 2021[1]; Bröde et al., 2023[2]; Kuklane et al., 2022[8]; Lunerová et al., 2023[9]; Młynarczyk et al., 2018[11]). Though modern digital human models for industrial work design (Spitzhirn et al., 2022[15]; Yin and Li, 2023[17]) include functional tests of vision, mobility, or biomechanical load with relevance for human testing (DIN EN 17558, 2023[3]), their application to PPE use conditions is rare.

Recognizing the increased awareness for simulation tools supporting PPE ergonomics, a session on 'Digital Human Models and Virtual Ergonomics of PPE Systems' was held at the 10^th^ European Conference on Protective Clothing (*ECPC2023*), 9-12 May 2023, in Arnhem, The Netherlands. While the contributions covered all six levels in Figure 1(Fig. 1) (Santos et al., 2023[14]), we like to feature two papers introducing novel methodologies in the scope of virtual ergonomics of PPE systems. The first study (Martinez-Albert et al., 2023[10]) described a protocol for testing the capacity of heat stress mitigation by cooling devices worn underneath protective clothing using a thermal manikin coupled with a human thermoregulation model, thus combining levels 2 and 3 (Figure 1(Fig. 1)). At the intersection of levels 3 and 4, the second methodical paper (Muenks et al., 2023[12]) considered issues in measuring the dynamic fit of firefighter clothing during typical activities utilizing high speed 4D body scanning with relevance to the ergonomic assessment of user mobility.

### Standardization perspective

In addition, an expert panel at *ECPC2023* discussed the role for standardization in strengthening the development and usage of virtual ergonomics tools for PPE application. The desiderata to standardization comprised all levels (Figure 1(Fig. 1)) ranging from material input data, over physiological assessment criteria to databases with field data for validation.

Although isolated use cases were identified (Bröde et al., 2023[2]; Kuklane et al., 2022[8]), there was consensus that transferring holistic virtual concepts, like modelling the biomechanical health risks of load carriage (Jäger, 2023[7]), to PPE use conditions will require further efforts in terms of work, time and budget.

## Declaration

### Ethical statement

Not applicable.

### Conflict of interest

The authors had co-developed the standard EN 17558:2023 as members of the former working group CEN/TC122/WG14-*Ergonomics of PPE Systems*.

### Acknowledgment

We thank the board of the European Society of Protective Clothing and the members of CEN/TC122/WG14 for supporting this work.
